# Shared germline-biased plasmablast clonotypes shape early neutralizing antibody responses during acute Chikungunya virus infection

**DOI:** 10.3389/fimmu.2026.1846926

**Published:** 2026-06-19

**Authors:** Kritika Dixit, Kaustuv Nayak, Manpreet Kaur Saini, Sofia Qamar, Prashant Bajpai, Elluri Seetharami Reddy, Sahil Ameer, Vikas Dhar Dubey, Muneeb Pervez, Sagar Kohli, Kamalvishnu Gottimukkala, Shipra Gupta, Naushad Khan, Deepti Maheshwari, Nawaz SM Akhtar, Yadya M. Chawla, Ramesh Chandra Rai, Rohit Sagar, Mohammad Islamuddin, Wajihul Hasan Khan, Nimisha Mishra, Ashok Kumar Patel, Sujatha Sunil, Rakesh Lodha, Vineet Jain, Pratima Ray, Kaja Murali-Krishna, Keshav Saini, Sanjeev Kumar, Anmol Chandele

**Affiliations:** 1 ICGEB-Emory Vaccine Program, International Centre for Genetic Engineering and Biotechnology, New Delhi, India; 2 Kusuma School of Biology Sciences, Indian Institute of Technology, New Delhi, India; 3 Department of Biotechnology, School of Chemical & Life Sciences, Jamia Hamdard, New Delhi, India; 4 Vector-Borne Diseases, International Centre for Genetic Engineering and Biotechnology, New Delhi, India; 5 Department of Pediatrics, All India Institute of Medical Sciences, New Delhi, India; 6 Department of Medicine, Hamdard Institute of Medical Sciences and Research (HIMSR), New Delhi, India; 7 Department of Pediatrics, Division of Infectious Diseases, Emory University School of Medicine, Atlanta, GA, United States; 8 Emory Vaccine Center, Emory University, Atlanta, GA, United States

**Keywords:** acute viral infection, Chikungunya virus, germline antibodies, human monoclonal antibodies, memory B cells, neutralizing antibodies, plasmablasts, single B cell analysis

## Abstract

**Introduction:**

Chikungunya virus (CHIKV) infection causes acute febrile illness and severe joint inflammation, with some patients progressing to chronic arthritis. Early isotype-switched neutralizing antibody (nAb) responses have been associated with protection from chronic arthritis, but the cellular and molecular features of these early responses remain poorly defined.

**Methods:**

We performed single-cell sorting of plasmablasts (PBs) from patients with acute CHIKV infection and generated 94 human monoclonal antibodies (mAbs). These were evaluated for binding to CHIKV-infected Vero cells and to purified CHIKV, neutralizing activity, isotype distribution, somatic hypermutation (SHM), and immunoglobulin gene usage. PB-derived antibodies were compared with CHIKV-specific memory B cell (MBC)-derived antibodies from seropositive individuals.

**Results:**

Over one-third of PB-derived mAbs recognized CHIKV-infected Vero cells, and most were class-switched. Among these, 12 bound to purified CHIKV, and 6 of them showed neutralizing activity. All PB-derived nAbs had germline-like sequences with little to no SHM, and four displayed shared clonotypic features, including a conserved `WEL' motif in the CDRH3 region. Conversely, CHIKV-specific MBC-derived nAbs showed diverse but often higher levels of SHM, consistent with affinity maturation.

**Discussion:**

Together, our findings suggest that early nAb responses generated during acute CHIKV infection are germline-biased with overlapping features suggestive of convergent clonotype responses, providing a cellular and molecular basis of the early protective humoral immunity.

## Introduction

Chikungunya virus (CHIKV) is a re-emerging arthropod-borne alphavirus that has caused repeated outbreaks across more than 100 countries over the past two decades (1–5). Infection typically presents as an acute febrile illness accompanied by arthritis and arthralgia (6, 7); however, a substantial proportion of patients progress to chronic inflammatory arthritis that can persist for months or even years. Despite extensive clinical and epidemiological investigation, the immunological mechanisms governing protection against the development of chronic disease remain unknown (6, 8).

Accumulating evidence indicates that antibody-mediated immunity plays a crucial role in controlling CHIKV infection and limiting chronic sequelae (9–14). Studies in animal models have demonstrated that B-cell-deficient hosts fail to clear virus from joint tissues, leading to persistent inflammation (15), whereas passive transfer of virus-specific antibodies improves disease outcome (10). In parallel, viral escape from defined neutralizing antibody (nAb) specificities, particularly those targeting the E2 glycoprotein, suggests a dynamic interplay between host immunity and viral evolution (10, 15). Consistent with these observations, we previously showed that patients who mount an early isotype-switched nAb response during the acute febrile phase are significantly less likely to develop chronic arthritis (16). Together, these findings implicate humoral immunity as a key determinant of both acute viral control and long-term disease progression.

While memory B cell (MBC) responses following recovery from CHIKV infection have been extensively characterized (13, 17–19), considerably less is known about plasmablasts (PBs), the transient antibody-secreting B cells that expand rapidly during active infection and generate the first wave of circulating antibodies. PBs provide a real-time snapshot of the ongoing humoral response and often precede the establishment of germinal center reactions and long-lived memory (20, 21). In many other viral infections, such as Dengue, Influenza, Ebola, or SARS-CoV-2, PB-derived antibodies exhibit diverse maturation states and functional capacities (21–23); however, the molecular features, isotypes, and neutralization potential of PB-derived antibodies during acute CHIKV infection remain poorly defined.

A detailed understanding of early PB responses is particularly relevant for CHIKV, as the early neutralizing activity has been associated with protection from chronic disease. In this study, we performed single-cell analysis of circulating PBs from individuals with laboratory-confirmed acute CHIKV infection and compared their antibody repertoires with CHIKV-specific MBCs from seropositive donors. Our findings show that early PB responses during CHIKV infection include antibodies that recognize infected cells and purified virus, some of which also neutralize the virus. These nAbs are germline-like, and four of them share clonotype features, including a conserved ‘WEL’ motif in the CDRH3 region, suggesting that the early nAbs that can appear in some patients during the acute phase are generated without requiring extensive somatic hypermutation (SHM). Our data also show that with progressive time, this primary infection eventually results in an affinity-matured MBC with multiple rounds of SHM, which may help sustain long-term immunity. These insights have important implications for understanding CHIKV antibody responses and for guiding vaccine strategies that aim to elicit protective antibodies.

## Results

### Acute CHIKV infection results in a transient and class-switched PB response

**Figure 1:**
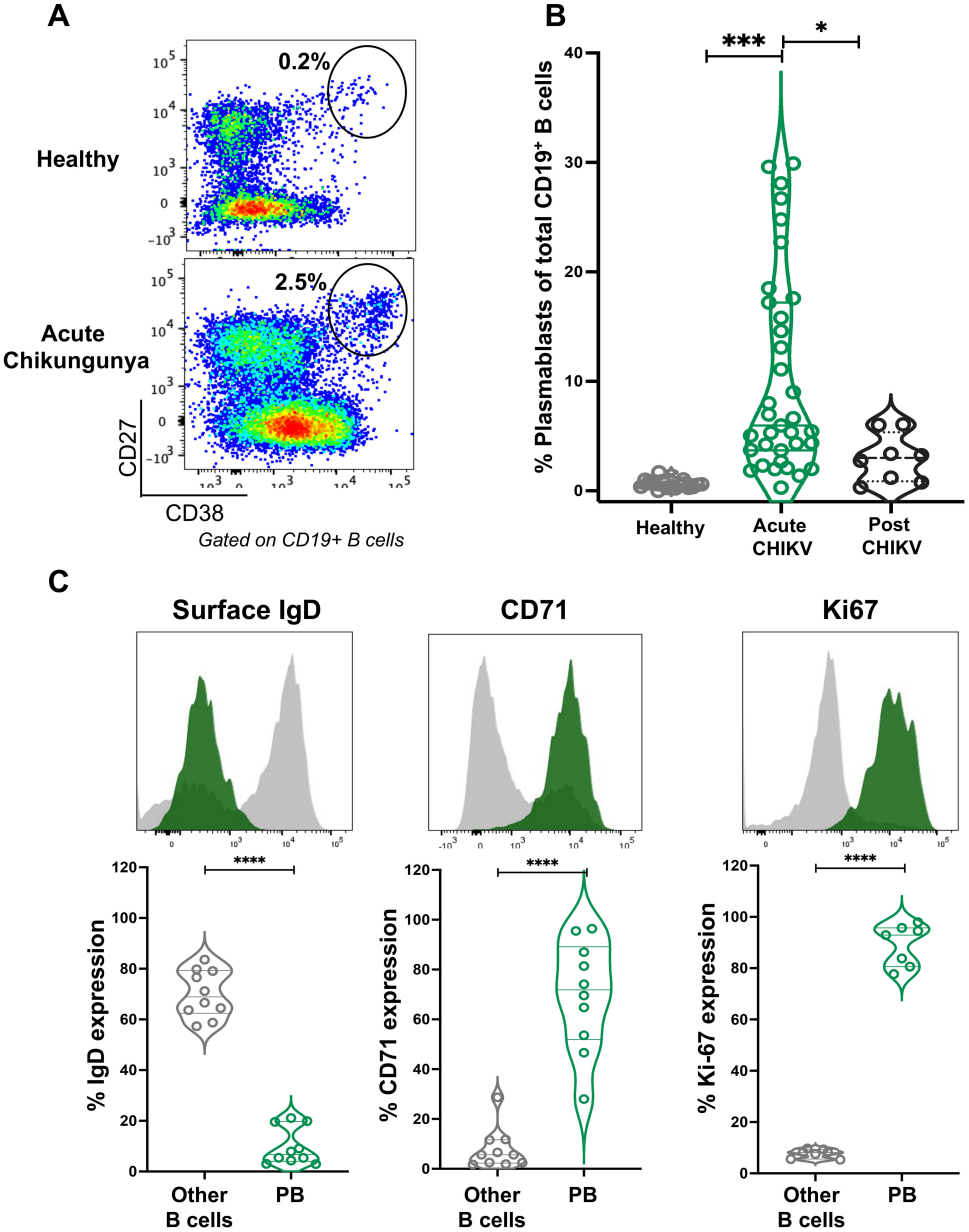
Acute CHIKV infection results in a transient and class-switched PB response. PBMCs from healthy controls and individuals with acute CHIKV infection were analyzed by flow cytometry. **(A)** Shows representative flow cytometry plots showing gating of PBs (CD3^-^CD20^-^CD19^int/+^CD27^++^CD38^++^ cells) in healthy donors and acute CHIKV–infected individuals. **(B)** The scatter graph shows the frequency of PBs expressed as a percentage of total CD19^+^ B cells in healthy controls (grey), CHIKV patients (green), and post-recovery (black). Statistical significance was determined using the Mann–Whitney U test, with p values denoted as follows: *p ≤ 0.05; **p ≤ 0.01; ***p ≤ 0.001; ****p ≤ 0.0001. **(C)** Overlay histograms of canonical phenotype markers is shown for PBs (green histogram) overlaid on other (non-PB) B cells (grey histogram). The graphs below each overlay histogram provide a summary of 9–10 CHIKV confirmed patients. Statistical significance was determined using the Mann–Whitney U test, with p values denoted as follows: ****p < 0.0001.

We first quantified PB responses in 35 patients with acute febrile illness and laboratory-confirmed CHIKV infection. Demographic details of these individuals are provided in **Table 1**. As controls, PBMCs from healthy individuals and from CHIKV-recovered individuals were also analyzed in parallel. PBs were phenotypically defined as CD3^-^CD20^-^CD19^int/+^CD27^++^CD38^++^ cells. We detected a clear PB response in many patients during the symptomatic phase, which was significantly higher than in non-febrile (healthy) individuals (**Figure 1A**). Inter-individual variation was evident, and the PB frequencies ranged from 0.2% to 30%, with an average expansion of ~9% that contracted in individuals who recovered from acute CHIKV (**Figure 1B**). These PBs expressed canonical markers of activation and proliferation (CD71 and Ki-67) and did not express surface IgD, indicating they were no longer naïve (**Figure 1C**). The PB expansion occurred among acute febrile patients who exhibited CHIKV-specific nAbs, as well as among those with undetectable CHIKV-specific nAbs in their plasma (**Supplementary Figure 1**). These data indicate that acute CHIKV infection induces a transient PB expansion consistent with a recent B cell activation.

**Table 1:** Shows characteristics of CHIKV confirmed patients in whom PB responses were studied.

Characteristics of Chikungunya confirmed acute patients (n = 35)	
Days post onset of fever (Avg, Range)	5 (2–7 days)
Age (Avg, Range)	38 (20–65 years)
Gender (M/F)	19/16
CHIKV PCR positive*	10
CHIKV IgM Units**	34 (2.2–74.1)
CHIKV IgG Units***	23.4 (1.7–80.8)

* in-house CHIKV specific PCR.

** Abcam CHIKV IgM ELISA cut off 11 units.

*** Abcam CHIKV IgG ELISA cut off 11 units.

### A significant fraction of PB clones are class-switched, show binding to CHIKV, and are capable of neutralizing the virus

We then examined the quality of antibodies produced by PBs during the acute febrile phase of CHIKV infection. Human monoclonal antibodies (mAbs) were generated following single-cell sorting of PBs from six CHIKV-confirmed patients with detectable PB responses. Patients 1–3 were CHIKV PCR positive with no nAbs in plasma, and patients 4–6 CHIKV PCR negative, CHIKV IgM and CHIKV nAb positive (**Supplementary Table 1****).** The sorting strategy is shown in **Supplementary Figure 2**. Since CHIKV infection is known to confer lifelong protective immunity, we also examined the presence of CHIKV-specific MBCs in buffy coat samples of healthy seropositive individuals who had recovered from acute CHIKV many months ago. For this, an MBC assay was performed. We found CHIKV-specific MBCs in most of these buffy coats, with heterogeneous frequencies ranging from 0.005% to 3.2% (mean: 1.4%) of total IgG-secreting B cells (**Supplementary Figure 3A**). Then, for comparison with the acute PB-derived mAbs, we also generated human mAbs from single-cell-sorted CHIKV-specific MBCs identified using whole CHIKV as an antigenic bait. This was performed using PBMCs from five individuals (**Supplementary Table 2**) who had detectable CHIKV nAbs and MBC frequencies. Sorting strategy is shown in **Supplementary Figure 3B**.

**Figure 2:**
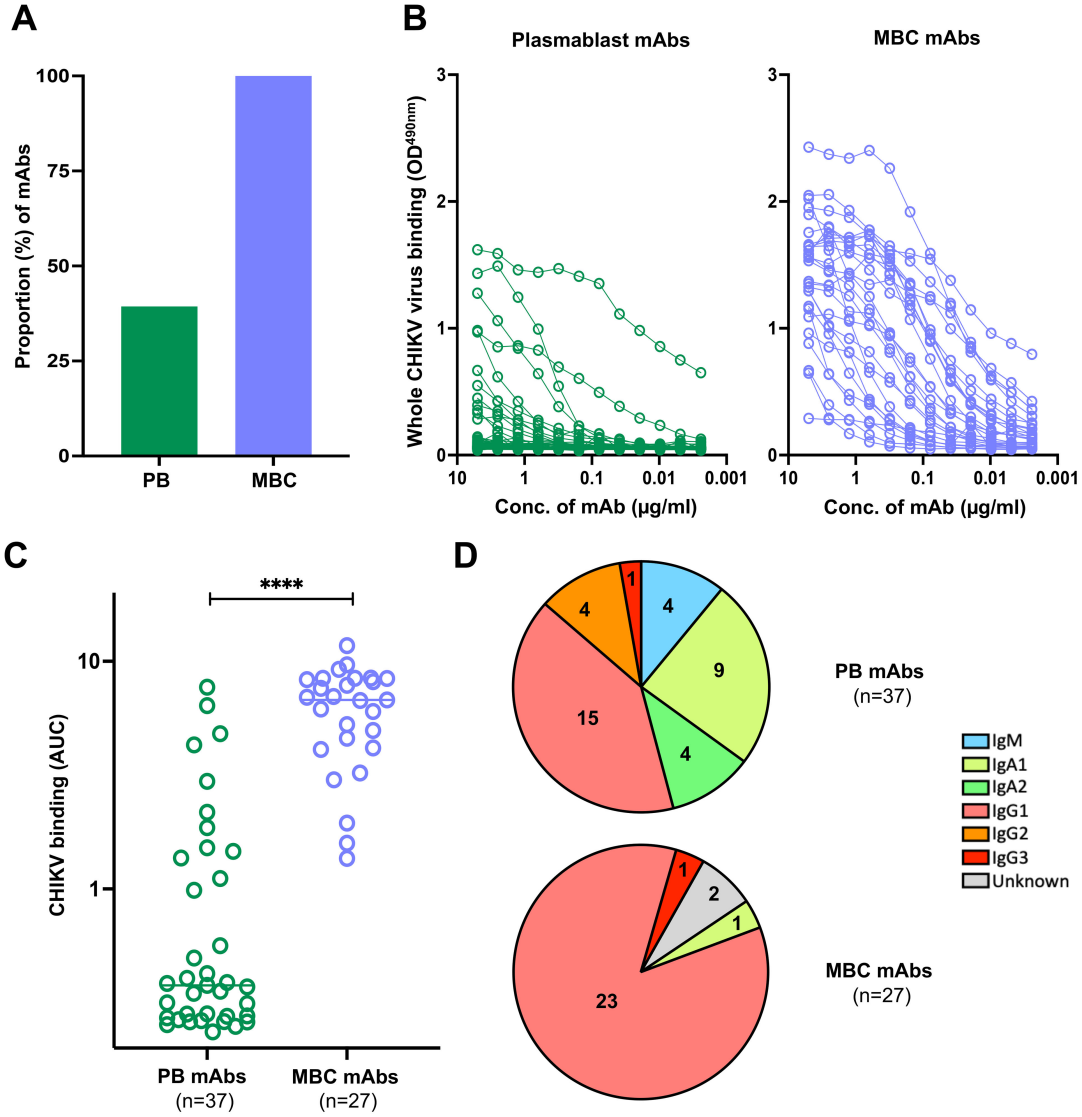
A significant fraction of PB clones is class-switched and show binding to CHIKV. Human mAbs were generated from single-cell sorted PBs (n = 94) and MBCs (n = 27) isolated from individuals with acute CHIKV infection and healthy individuals, respectively. **(A)** Proportion of CHIKV-binding mAbs identified using CHIKV Vero cell-infected lysates, showing reactivity among PB-derived mAbs (green; 37/94) and MBC-derived mAbs (violet; 27/27). **(B)** ELISA binding curves of representative PB-derived (green; n = 12) and MBC-derived (violet; n = 27) mAbs to plates coated with UV-inactivated CHIKV virions. **(C)** Area-under-the-curve (AUC) analysis of ELISA binding responses demonstrates significantly greater apparent binding strength of MBC**-**derived mAbs compared with PB-derived mAbs. Statistical significance was determined using the Mann-Whitney U test, with p values denoted as; ****p ≤ 0.0001 Results are depicted as median and interquartile ranges. **(D)** Immunoglobulin isotype distribution of PB-derived mAbs, indicating that the majority of antibodies generated during the acute febrile phase of CHIKV infection are already class-switched at the single-cell level.

We then studied the proportion of PB-derived mAbs that bound to CHIKV using two different methods: flow cytometry-based binding assays and indirect ELISA. We found that of the total 94 PB mAbs generated, 37 mAbs showed detectable CHIKV binding by flow cytometry (**Figure 2A**, green bar). Of these, 12 mAbs also showed binding by ELISA (**Figures 2B**, left), in which the coating antigen was fixed CHIKV, suggesting that a subset of PB-derived antibodies recognized antigens that were detectable in infected cells but not efficiently represented in purified virions. These PB-derived mAbs also had lower apparent binding strengths as calculated by area under the curve (AUC) (**Figure 2C**) in comparison to MBC-derived mAbs (n=27) that were all CHIKV-reactive (**Figure 2A**, blue bar) and showed statistically significantly higher binding affinities to CHIKV virus (**Figures 2B, C** right, and blue).

Interestingly, only a small fraction of CHIKV-reactive PB mAbs (n=37) were of the IgM isotype, with the majority already class-switched to IgA or IgG isotypes, indicating that PBs responding to primary CHIKV infection can undergo rapid class switching during the acute febrile phase (**Figure 2D**, **Supplementary Figures 4A, B**, left). These class-switched isotypes were not restricted to patients with detectable serum nAb titers and were observed in all individuals from whom PBs were sorted, and mAbs were generated (**Supplementary Figure 5**). In comparison, all MBC-derived mAbs (n=27) were mostly IgG1, with one IgA1 and one IgG3 clone; though three clones could not be isotyped (**Figure 2D**, **Supplementary Figures 4A, B**, right).

**Figure 3:**
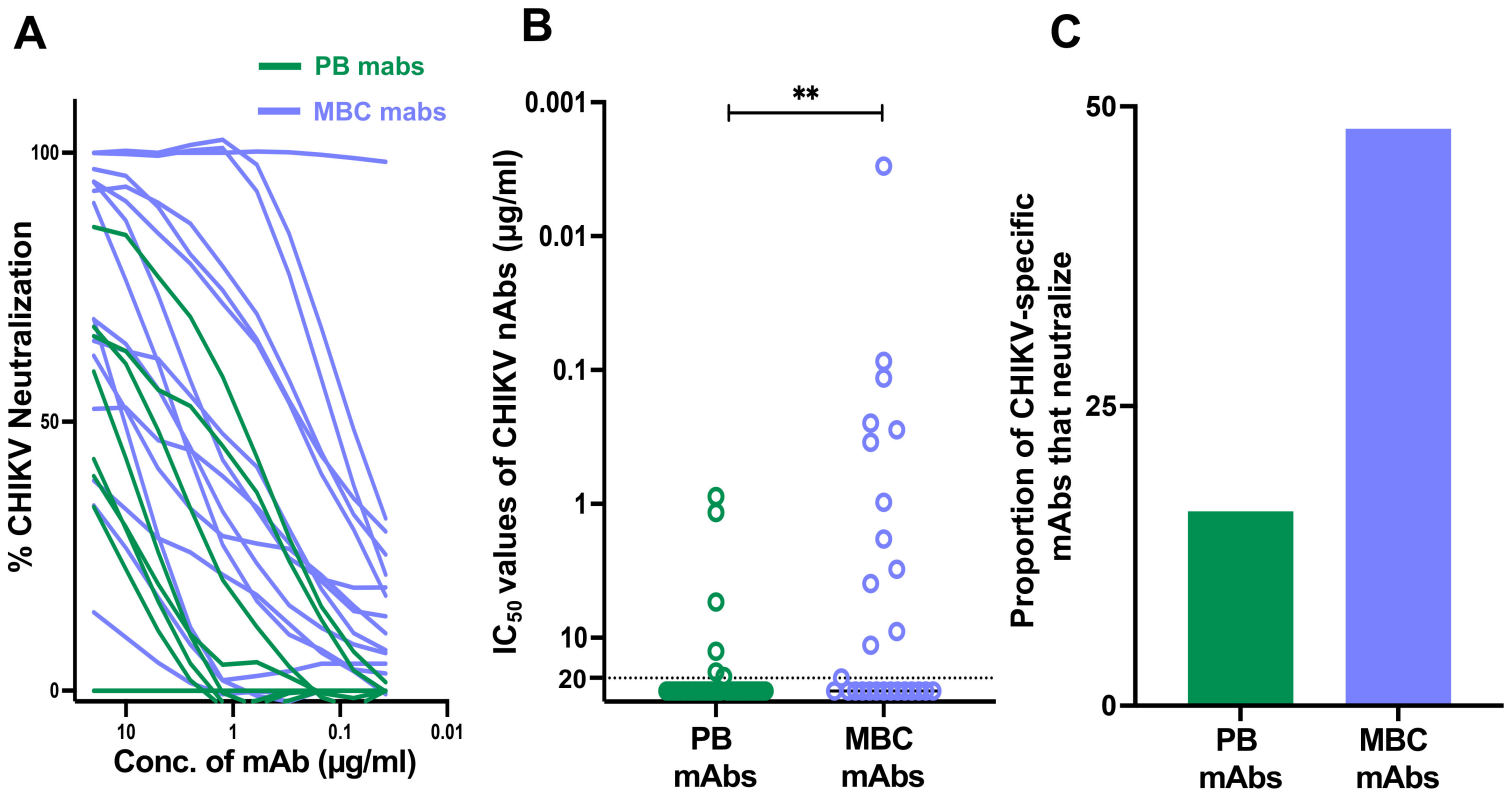
A small proportion of PB-derived mAbs are able to neutralize live CHIKV. Neutralization potency of CHIKV-reactive mAbs was assessed *in-vitro* using PRNT assays **(A)** Neutralization curves showing inhibition of live CHIKV infection by PB-derived mAbs (green lines) and MBC-derived mAbs (blue lines) across increasing antibody concentrations. **(B)** The scatter plot shows calculated IC_50_ values of PB- and MBC-derived nAbs. Statistical significance was determined using the Mann–Whitney U test, with p value of **p < 0.01. Results are depicted as median and interquartile ranges. Samples that do not neutralize at the limit of detection at 50% are shown as a dotted line. **(C)** Bar graph shows proportion of PB (6/37) and MBC-derived (13/27) mAbs exhibiting detectable CHIKV neutralization at antibody concentrations ≤20 µg/ml.

Next, we determined how many of the 37 PB-derived CHIKV-infected cell reactive mAbs and how many of the 27 MBC-derived CHIKV-specific mAbs were also capable of neutralizing CHIKV. To this end, we performed a live virus-based plaque-reduction neutralization test (PRNT) and determined the PRNT_50_ for each mAb (**Figure 3A**). We found that at least 6 (16.2%) of the 37 PB-derived mAbs showed detectable neutralizing activity against CHIKV. On the other hand, 13 of the 27 mAbs derived from MBCs (48.1%) neutralized CHIKV. Thus, the neutralizing capacity of mAbs derived from PBs was modest, both in terms of the frequency of the neutralizing clones as well as the neutralizing potency, as compared to MBC-derived mAbs (**Figures 3B, C**). Notably, all six nAbs within the PB-derived mAbs originated from patients 4 and 5, both of whom exhibited high serum nAbs titers early during the acute CHIKV phase. Interestingly, the MBC-derived mAbs from longitudinal sampling of patient 1, who did not show neutralizing activity during the acute febrile phase, did show neutralizing activity. Importantly, one of these MBC-derived mAbs obtained from longitudinal sampling of patient 1 at >6 months post-recovery from this primary acute infection showed exceptionally high potency, with an IC_50_ measurable only upon dilution to 37.5 picograms per ml. This antibody reduced *in vivo* viral titers as well as decreasing footpad swelling and overall morbidity in a mouse model of CHIKV infection (**Supplementary Figure 6**).

Together, these data show that PB-derived antibodies during acute CHIKV infection are largely class-switched but display heterogeneous antigen recognition and limited neutralizing activity. In contrast, MBC-derived antibodies are uniformly virus-reactive and enriched for higher-affinity, more potent neutralizing clones.

### Acute CHIKV PB-derived mAbs that neutralize the virus mostly exhibit germline sequences

To understand the emergence of CHIKV nAbs from PBs during the acute febrile phase of primary infection, when germinal center reactions are presumed to be limited, we examined the somatic hypermutation (SHM) profiles of the variable regions of PB and MBC-derived mAbs. Overall, PB-derived mAbs exhibited lower levels of SHM with an average of 4.06% in the heavy chain (HC, range: 0–13.51%) and 2.75% in the light chain (LC, range: 0–9.15%) compared to MBC derived mAbs that showed heterogeneous SHM levels, with HC mutation frequencies ranging from 2.5% to 13% (average: 5.95%) and LC mutations ranging from 1.3% to 11.26% (average: 3.5%) (**Supplementary Figure 7A**).

**Figure 4:**
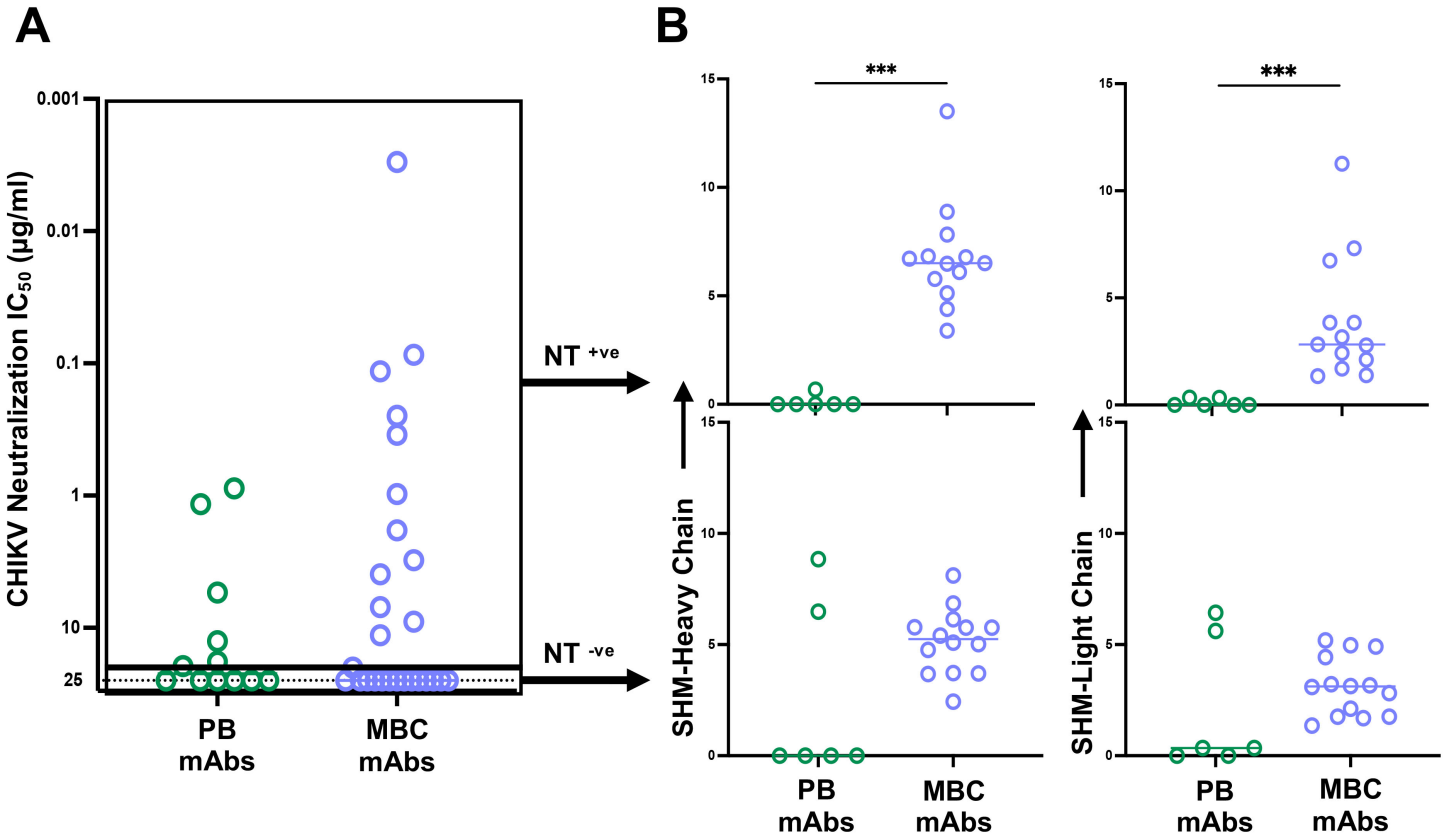
Acute CHIKV PB-derived mAbs that neutralize the virus mostly exhibit germline sequences. Immunoglobulin gene features of CHIKV-specific mAbs derived from PBs and MBCs were analyzed. **(A)** The scatter graph delineates nAbs (top box) and non-nAbs (bottom box) in both PBs and MBCs that were ELISA reactive (from **Figure 2B**). Samples that do not neutralize at the limit of detection at 50% are shown as a dotted line. **(B)** The right panel shows percent SHMs in heavy-chain nAbs (top) and non-nAbs (bottom) derived from PBs and MBCs. Left panel provides percent SHMs in light chain of PB- and MBC-derived nAbs (top) and non-nAbs (bottom). Statistical significance was determined using the Mann–Whitney U test, with p value of ***p < 0.001. Results are depicted as median and interquartile ranges.

Strikingly, when all mAbs that were ELISA reactive in PB (n=12) and MBC (n=27) were stratified by neutralizing potency, a clear distinction emerged. While MBC-derived mAbs continued to display heterogeneous SHM irrespective of neutralization capacity, all six PB-derived nAbs were completely germline-like, with four exhibiting zero accumulated mutations in both HCs and LCs (**Figures 4A, B**). This observation became even more profound when all CHIKV-specific mAbs from the PB compartment (n=37) were studied, while PB mAbs overall showed accumulation of SHM, such mutations were completely absent in the neutralizing clones (**Supplementary Figure 7B**).

These findings show that early neutralizing activity during acute CHIKV infection can be mediated by germline-like antibodies, consistent with previous clinical studies associating early neutralizing responses with protection from chronic arthritis (9, 24, 25). Interestingly, these near-germline features, although not absolute, were also evident in PB mAbs that showed binding but did not neutralize, suggesting that germline configuration alone is sufficient to confer recognition of CHIKV structural proteins.

Together, these observations suggest that the early PB response elicited during the acute febrile phase includes minimally mutated antibodies with features consistent with rapidly induced humoral responses, perhaps including extrafollicular pathways.

### PB-derived mAbs that neutralize CHIKV predominantly showed shared clonotypes

**Figure 5:**
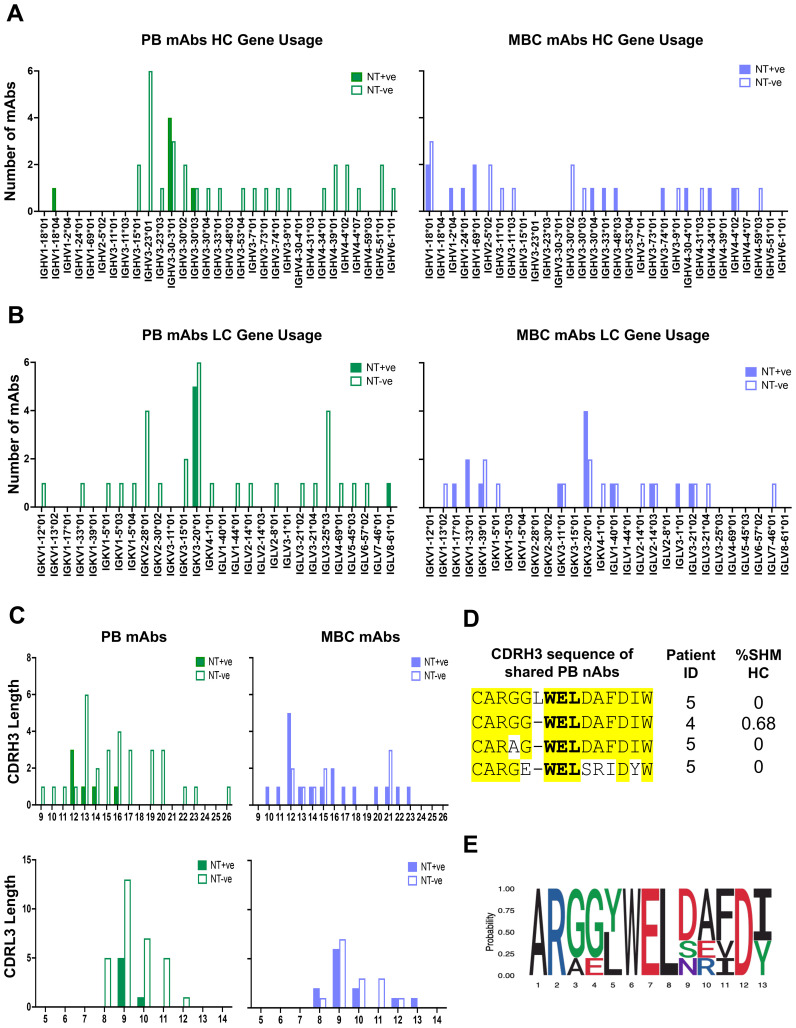
PB-derived mAbs that neutralize CHIKV predominantly showed shared clonotypes. **(A)** Bar graph shows differences in heavy chain gene usage in nAbs and non-nAbs from PBs (left) and MBCs (right). **(B)** The bar graph shows differences in light-chain gene usage in nAbs and non-nAbs from PBs (left) and MBCs (right). **(C)** Bar graph shows differences in lengths of CDRH3 and CDRL3 in nAbs and non-nAbs from PBs (left) and MBCs (right). **(D)** Alignment of the CDRH3 sequences with subject IDs of the top four PB-derived nAbs that belong to shared clonotypes, with their corresponding percent SHM in the heavy chain. **(E)** The sequence logo representation visualizes position-specific residue usage across CDR3 aligned with the 6 neutralizing PB mAbs.

The discovery of PB-derived neutralizing antibodies (nAbs) with germline-like sequences prompted detailed analysis of the immunoglobulin gene usage and CDR3 features of PB-derived CHIKV mAbs. PB-derived nAbs were dominantly encoded by the IGHV3-30-3*01 heavy chain gene and IGKV3-20*01 light chain gene (**Figures 5A, B**, left). Analysis of CDR3 regions showed that CDRH3 and CDRL3 lengths of 12 and 9 amino acids (aa), respectively, were most frequently observed (**Figure 5C**). Notably, the top four PB-derived nAbs displayed sequence convergence, shared a conserved ‘WEL’ motif in CDRH3, and belonged to clonotypes encoded by the IGHV3-30-3*01, IGHD1-26*01, and IGKV3-20*01 gene combination (**Figures 5D, E**; Supplementary Sheet). Three of these shared clones originated from the same subject, while one was identified from another subject. Interestingly, PB-derived neutralizing mAbs also showed significantly shorter length of CDRH3 compared to non-nAbs (average: 13 aa vs. 16 aa; **Supplementary Figure 8A**), whereas length of CDRL3 between nAbs and non-nAbs were comparable (average: 9.1 aa vs. 9.4 aa; **Supplementary Figure 8B**).

In contrast, MBC-derived nAbs exhibited a more diverse heavy and light chain gene usage, with the most potent clone encoded by IGHV4-30-4*01 (**Figures 5A**, right; supplemental sheet) and heterogeneous light chain utilization (**Figures 5B**, right). Unlike PB-derived nAbs, MBC-derived nAbs did not display germline-like features or clonal convergence. CDRH3 lengths in MBC-derived neutralizing mAbs had an average length of 14.6 aa, which were shorter than non-neutralizing MBC antibodies (17.2 aa; **Supplementary Figures 8A, B**), which was similar to what was observed for PB-derived mAbs, whereas the length of CDRL3 between neutralizing mAbs and non-neutralizing antibodies was comparable (average: 9.5 aa vs. 9.7 aa; **Supplementary Figure 8B**).

Taken together, these findings demonstrate that PB-derived CHIKV nAbs are characterized by restricted immunoglobulin gene usage, germline-like sequences, and convergent clonotypic features, including conserved motifs in CDRH3 and shorter CDRH3 lengths. In contrast, MBC-derived nAbs display broader gene diversity and lack evidence of clonal convergence, though neutralizing function was evident, with nAbs exhibiting shorter CDRH3 lengths. These results suggest that early PB responses preferentially generate convergent nAbs, whereas MBC responses reflect a greater diversification and maturation.

## Discussion

Early nAbs detected during acute CHIKV infection have been associated with protection against progression to chronic arthritis (24, 25), yet the cellular origins and molecular features of these responses have remained poorly defined. In this study, we provide a single-cell, functional dissection of the human B cell response to primary CHIKV infection, with a particular focus on the early PB compartment during the acute febrile phase. By generating and characterizing mAbs from circulating PBs and comparing them with antibodies derived from CHIKV-specific MBCs, we reveal fundamental qualitative differences between early and late humoral responses. Our findings demonstrate that acute CHIKV infection induces a robust, class-switched PB response that includes germline-biased nAbs belonging to shared clonotypes, consistent with an extrafollicular origin. In contrast, the MBC compartment is enriched for affinity-matured antibodies with greater neutralization potency, supporting a model in which early germline-encoded responses provide rapid antiviral defense that is subsequently refined through germinal center selection into durable immune memory.

A key initial finding of this study is that most febrile individuals with laboratory-confirmed CHIKV infection mount a measurable PB response during the acute phase. However, it was not as massive as previous studies reported in Dengue virus infection (21, 26) and the timing and minimally mutated nature of the PB responses observed here are reminiscent of rapidly induced antiviral plasmablasts responses described in influenza, dengue, and SARS-CoV-2 infection, including responses proposed to involve extrafollicular activation (21–23). Although inter-individual variability was evident, likely reflecting differences in viral burden, sampling time points, and other host factors, these data indicate that PB expansion is a reproducible feature of early CHIKV infection.

Notably, analysis of the human mAbs generated from single-cell-sorted PBs showed that only a subset of PB-derived antibodies recognized CHIKV structural proteins and were ELISA positive, revealing heterogeneity within the acute response, with a proportion of PB clones recognizing antigens detectable in infected cells but not efficiently represented in purified virions. This may include recognition of non-structural proteins, although direct epitope mapping will be required to define antigen specificity. However, these observations align with studies reporting antibody responses against CHIKV non-structural proteins in both human sera and CHIKV-infected macaques (27, 28).

One of the most striking observations of this study is that all nAbs derived from PBs exhibited germline-like or near-germline sequences, with minimal or no detectable SHM. While this finding is in odds with the prevailing view that potent antiviral neutralization necessarily requires extensive affinity maturation, similar germline-biased neutralizing responses have been reported for Influenza virus and SARS-CoV-2 and have been associated in previous studies with rapidly induced antiviral responses, including those proposed to involve extrafollicular activation (29–31). In the context of CHIKV, such early neutralization may be particularly important for limiting viral dissemination to musculoskeletal tissues for constraining subsequent inflammatory pathology (18). Notably, the patients from whom the majority of binding and neutralizing PB-derived mAbs were generated in this study did not subsequently develop long-term arthritis. While the present study was not designed to directly test clinical protection, this observation is consistent with previous reports associating early binding and neutralizing antibody responses with favorable disease outcome. This observation is consistent with previous reports showing that early binding and nAb responses are associated with protection against chronic symptoms (24, 25).

PB-derived nAbs displayed pronounced clonotypic convergence, conserved CDRH3 motifs, and shorter CDRH3 lengths, consistent with strong structural constraints imposed by dominant neutralizing epitopes. These features are consistent with convergent antibody responses and suggest that similar binding geometries may be preferentially selected during early infection (32–34). The enrichment of shorter CDRH3 regions among PB-derived neutralizers, relative to non-nAbs, further supports a model in which specific structural configurations are preferentially recruited into the early antiviral response (30, 35). However, the number of donors analyzed in this study was limited, and thus the conclusions are based on a relatively small number of PB-derived neutralizing antibodies identified; therefore, a broader generalization regarding shared or convergent germline-biased responses should be interpreted cautiously and will require validation in larger cohorts.

Interestingly, preferential usage of IGHV3–30 gene family has also been independently observed in other CHIKV antibody studies. Malonis et al. previously described a potent CHIKV-reactive monoclonal antibody encoded by IGHV3-30, although paired with a distinct light chain (18). Similarly, recent studies examining antibody responses following CHIKV vaccination have reported over-representation of IGHV3–30 gene usage among CHIKV-reactive antibodies (36). Together with our findings, these observations suggest that IGHV3-30-encoded responses represent a recurrent characteristic gene for CHIKV recognition and neutralization across both natural infection and vaccination settings.

In contrast, MBC-derived antibodies uniformly recognized CHIKV and exhibited greater apparent binding strength, higher neutralization potency, and extensive SHM, underscoring the central role of germinal center selection in shaping durable humoral immunity. However, not all memory-derived antibodies were neutralizing, indicating that entry into the memory pool is not driven solely by antiviral potential. Rather, the MBC compartment appears to preserve a functionally diverse repertoire while selectively enriching for highly optimized neutralizers. The identification of MBC-derived antibodies capable of conferring *in-vivo* protection further highlights the importance of affinity maturation in refining antiviral efficacy.

A limitation for some of this analysis is that PB antibodies originally identified as IgM or IgA isotypes were recombinantly expressed as IgG1 molecules to enable standardized functional comparisons across clones. As antibody valency and Fc architecture can substantially influence avidity, antigen engagement, and neutralization potency, the functional activity of the native antibody isotypes may differ from that observed in the recombinant IgG1 format. Lastly, while PBs and MBCs were sampled from distinct compartments and time points, limiting direct longitudinal lineage tracing, the combined molecular and functional analyses provide indirect evidence for a sequential maturation process. Early germline-biased responses appear to prioritize speed and structural convergence, whereas later germinal center reactions promote diversification, affinity optimization, and long-term stability. However, while the molecular features of PB-derived antibodies are consistent with rapidly induced humoral responses, the present study does not directly define the anatomical or cellular origin of these responses. Therefore, interpretations regarding extrafollicular versus germinal center pathways should be considered cautiously. Future longitudinal studies integrating single-cell sequencing with temporal sampling will be valuable for resolving clonal relationships between early and late compartments.

Taken together, this study delineates fundamental qualitative differences between early PB-derived and MBC-derived antibody responses to CHIKV. Our findings provide molecular context for previous clinical observations linking early nAbs with favorable outcomes and refine their interpretation by demonstrating that such activity can originate from minimally mutated repertoires. From a translational perspective, these results highlight the importance of vaccine strategies that engage germline B cell receptors to elicit rapid protection while simultaneously supporting robust germinal center responses for durable immunity.

## Materials and methods

### Human subjects

Acute febrile patients with CHIKV infection-like symptoms were recruited at the out-patient department of Jamia Hamdard Hospital, after obtaining informed consent. Blood samples were collected in cell preparation tubes (CPT) (Becton Dickenson, Cat# 362761) and transported immediately to the lab. CHIKV infection was confirmed by a combination of methods, including reverse transcription (RT)-PCR, CHIKV IgM capture ELISA (AbCam Cat #ab108717). Patients positive for CHIKV IgM and/or PCR and negative for dengue PCR, NS1, IgM/IgG were included in this analysis. Ethical clearance was obtained from all participating institutions and the ICGEB ethical clearance number is ICGEB/IEC/2012/01, version 2. For CHIKV recovered subjects, either patients were requested for an additional blood draw post recovery, or otherwise discarded, and medically not used Buffy coats were collected from the Department of Transfusion Medicine, AIIMS (New Delhi, India), and their plasma was tested for their CHIKV binding and nAb titers.

### PBMC and plasma isolation

Peripheral blood mononuclear cells (PBMCs) and plasma were isolated from the collected blood in the CPT as outlined previously (16). Briefly, CPT tubes were centrifuged at the room temperature (RT), 1500 x g for 25 minutes without brake at RT. The topmost layer of plasma was transferred into several aliquots in cryovials and stored immediately at -80 °C and utilized for CHIKV ELISA and neutralizing assays. The PBMC layer above the gel plug was transferred to a sterile 15 mL conical tube and washed twice with 1X phosphate-buffered saline (PBS) (HiMedia #TL1099). Residual red blood cells (RBCs) were lysed using RBC lysis buffer, followed by additional PBS washes. PBMCs were cryopreserved in freezing medium consisting of 90% fetal bovine serum (FBS) and 10% dimethyl sulfoxide (DMSO) and stored in liquid nitrogen until further use.

### Enumeration of PB responses

PBMC with sufficient recovery were thawed, thoroughly washed and stained for flow cytometric analysis. Cells were incubated with the fixable viability dye eFluor 780 (eBioscience, Cat# 65-0865-14) to exclude dead cells. Surface staining was performed using fluorochrome-conjugated antibodies against CD19 (BD, Cat# 562294), CD20 (BD, Cat# 347647), CD38 (BD, Cat# 562288), and CD27 (eBioscience, Cat# 17-0249). Cells were incubated with antibody cocktail on ice for 30 minutes, washed with FACS buffer (0.25% FBS in PBS), and fixed using BD Cytofix for 10 minutes on ice. After washing, cells were resuspended in FACS buffer for acquisition on a flow cytometer. PBs were defined phenotypically as CD3^-^CD20^-^CD19^int/+^CD27^++^CD38^++^ cells.

### Immunophenotyping of PB responses

For extended immunophenotyping, the PBs were also stained with CD71 (Biolegend, Cat# 334108) and IgD antibodies (BD, Cat# 555778). For intracellular Ki-67, cells were first surface stained, followed by fixation and permeabilization using Cytofix/Cytoperm buffer (BD), followed by staining with anti-Ki67 (BD, Cat# 561283) antibody for 60 min in Perm/Wash buffer (BD, Cat# 554723), washed and acquired.

### Identification of CHIKV seropositive subjects

CHIKV seropositivity was determined using a plaque reduction neutralization test (PRNT)-based assay using plasma samples from buffy coats (n=121) as described previously with a few modifications (16). Briefly, plasma samples were heat-inactivated and serially diluted (two-fold) and co-incubated with 50 plaque forming units (PFU) of virus for 1 h at 37 °C. Following incubation, the mixture was plated on 20,000 vero cells in a 96-well plate, overlaid with 2% (wt/vol) methylcellulose (Sigma; #M0512-2506) and further incubated for 36 hrs. The plaques were revealed using crystal violet staining to visualize plaques. Neutralization titers (IC_50_) were calculated, and samples showing ≥50% neutralization at a plasma dilution of 1:50 were classified as seropositive.

### Memory B cell assay

Standard polyclonal MBC assay was performed on a subset of exposed individuals that had high titers of CHIKV-specific nAbs. Briefly, 0.5x10^6^ PBMCs were stimulated with pokeweed mitogen, Staph A and CpG in a 24-well plate for 5 days followed by CHIKV-specific ELISpot assays. Samples with high frequency of MBCs were selected for single B cell sorting using whole CHIKV as antigenic bait.

### Baiting of CHIKV specific memory B cells in CHIKV immune subjects

PBMCs from a subset of the CHIKV immune individuals were stained for CHIKV-specific MBCs. For this, 10x10^6^ PBMCs were incubated with 1 µg purified CHIKV virus for 1 hr, followed by extensive washing. CHIKV-specific B cells were detected by staining with CD3, CD19, CD20, CD27 and fluorochrome conjugate anti-CHIKV antibody. After thoroughly washing, the cells were immediately acquired on a flow cytometer. Lymphocytes that were CD19^+^CD20^+^CD27^+^ and CHIKV-antigen positive, where as negative for IgD and CD3 were considered as CHIKV-specific MBCs.

### Single cell sorting of PBs and CHIKV specific memory B cells

Extensively washed PBMCs were stained with fluorochrome labeled antibodies and fixable viable dye eFluor 780 (eBioscience, Cat# 65-0865-14) for dead cells exclusion. For PBs, cells were stained with CD19 (BD, Cat# 562294), CD20 (BD, Cat# 347647), CD38 (BD, Cat# 562288) and CD27 (ebioscience, Cat# 17-0249); and for CHIKV specific MBCs CD19 (BD, Cat# 562294), CD20 (BD, Cat# 347647), IgD (BD, Cat# 555778) and CD27 (ebioscience, Cat# 17-0249), and in-house anti-CHIKV mAb conjugated with Alexa 647. The antibody cocktail solution was prepared and added onto the cells and kept on ice for 30 mins. The cells were then thoroughly washed and resuspended in PBS containing 2% FBS, and then single cell sorted in a 96-well plate on a BD FACS Aria III (BD) with high forward-scatter gates to account for the larger blasting effector lymphocytes. For PBs, CD19^+/int^ CD20^-^ CD27^++^ CD38^++^ and for CHIKV specific MBCs CD19^+^ CD20^+^ CD27^+^ IgD^-^ and CHIKV virus positive were single cell sorted in 96-well plate containing 10 µl of RNase-inhibiting RT-PCR catch buffer allowing for immediate lysis of the single cells and preservation of the RNA. The plates were immediately sealed with a micro seal foil label and placed on dry ice until the cell sorting finished then plates were placed in a -80 °C freezer.

### Single B cell RT-PCR, amplification, cloning, and expression of antibody variable genes

The amplification of variable heavy (VH) and light chain (VL) antibody genes was performed as previously described (37). Briefly, frozen 96-well plates containing sorted single B cells was first thawed on ice, followed by cDNA synthesis using 1 µl of 200 U/well Superscript III reverse transcriptase (Invitrogen), 1 µl of 10 mM deoxyribonucleotide triphosphate (dNTP) mix (Thermo Fisher), 0.5 µl of 50 ng/ml random hexamers (Thermo Fisher), and 0.5 µl of oligo(dT) (Thermo Fisher) under the following cycling parameters: 25 °C for 10 min, 42 °C for 10 min, 50 °C for 50 min, 55 °C for 10 min, and 85 °C for 5 min, with a hold at 4 °C. Next, the first-strand cDNA was amplified by two-step nested PCR. In the first step, 0.4 µl of the mixture of 5 µM IgH, Igλ, or Igκ chain-specific primers were used as previously described for the amplification of antibody variable genes of heavy and light chains in a final reaction volume of 25 µl using 0.3 µl of DreamTaq DNA polymerase (5 U/µl) (Thermo Fisher), 3 µl of first-strand cDNA, and 0.3 µl of 10 mM dNTP mix (Thermo Fisher) under the following cycling parameters: 95 °C for 5 min and 35 cycles of 95 °C for 30 s, 50 °C or 52 °C for 60 s, 72 °C for 1 min, and 72 °C for 10 min, with a hold at 4 °C. Next, 0.4 µl of the mixture of 5 µM IgH, Igλ, or Igκ chain cloning primers were used for the second round of the PCR in a final volume of 25 µl using 0.3 µl of DreamTaq DNA polymerase (5 U/µl) (Thermo Fisher), 0.5 to 2 µl of first-round PCR product, and 0.3 µl of 10 mM dNTP mix (Thermo Fisher) under the following cycling parameters: 95 °C for 5 min, followed by 38 cycles of 95 °C for 30 s, 55 °C or 60 °C for 60 s, 72 °C for1 min, and 72 °C for 10 min, followed by a hold at 4 °C. The nested PCR products were gel purified on 1.2% agarose gels and sent for sequencing. The variable gene sequence was translated using ExPASy and IMGT, and productive heavy and light chains pairs were cloned into AbVec IgG vectors using restriction enzyme digestion (Age1 and Sal1 for heavy chains; Age1 and BsiW1 for kappa chain; Age1 and Xho1 for lambda chain) and ligation, followed by transformation in DH5 *E. coli* cells. Positive clones were detected on ampicillin LB plates and plasmid DNA was isolated using plasmid isolation mini kit (Qiagen 27106). After confirmation of sequence, paired heavy and light chains were co-transfected into expi-293 cells, and antibody was purified using Protein A (Pierce; 20399) from the culture supernatant after 4–5 days.

### Somatic hypermutation analysis

Antibody variable heavy (VH) and variable light (VL) chain region sequences generated from single-cell sorted PBs and MBCs were analyzed using IMGT V-QUEST (38–40). SHM of the VH and VL regions refer to the total number of silent and replacement mutations between framework region 1 (FR1) and complementarity-determining region 3 (CDR3).

### CHIKV-specific ELISA

CHIKV binding antibodies in plasma and mAbs were assessed by ELISA using the following protocol. ELISA plates were coated overnight at 4 °C with 2 µg/ml of in-house purified UV inactivated whole CHIKV virus antigen in 1X PBS (pH 7.2). The next day, plates were washed three times with ELISA buffer (1X PBS + 0.05% Tween-20) and blocked for 1.5 hr at RT with 1% BSA. Then, either a 1:2 serial dilution of plasma or purified mAbs at a starting concentration of 5 µg/ml were added for 1 hr. Following a through wash with ELISA buffer the signal was revealed with anti-human IgG secondary antibody conjugated with horseradish peroxidase (HRP) (Jackson ImmunoResearch Labs, #109-036-098) followed by o-phenylenediamine (OPD) substrate (Sigma, #P8787) in 0.05M phosphate-citrate buffer (Sigma, #P4809) pH 5.0, containing 0.012% hydrogen peroxide (Fisher Scientific, #18755). Absorbance was measured at 490 nm and plasma antibody titers or area under curve of purified mAbs was calculated.

### Plaque reduction neutralization test

Plaque reduction neutralization test (PRNT) was performed to a**ss**ess plasma neutralization and neutralization of purified mAbs. For this, 30,000 Vero cells/well (clone E6) were seeded in a 96-well flat bottom plate overnight. Serially 2x diluted (1:25 starting dilution) heat-inactivated plasma samples or CHIKV mAbs (20 µg/ml starting concentration) were incubated with 50 PFU of CHIKV 181/25 clone virus for 1 hour at 37 °C with 5% CO_2_. The virus-plasma/antibody mixture was transferred onto Vero cells and were incubated for 1h at 37 °C with 5% CO_2_ followed by an overlay of 2% (wt/vol) methylcellulose (Sigma; #M0512-2506) containing ciprofloxacin and amphotericin B overlay. Cells were washed after 1 day of incubation at 37 °C with 5% CO_2_ with PBS and fixed for 1 hr at RT with 10% paraformaldehyde prepared in 1X PBS. After fixation, cells were washed with 1X PBS and stained with 0.1% crystal violet dye prepared in 30% methanol for 15 mins at RT. Plaques were scored on an ELISpot reader (CTL) and IC_50_ values were calculated using nonlinear regression method (curve fit) on prism software.

### *In vivo* analysis of CHIKV-specific neutralizing mAb

C57BL/6 mice were administered 20 µg/ml of purified CHIKV-specific MBC-derived mAb intravenously, followed by footpad infection with 1X10^6^ PFU of live CHIKV (ECSA strain). Viral titers were assessed, and cycle threshold values were determined by quantitative RT-PCR as described previously (41). Morbidity was scored based on multiple parameters, including changes in body weight, clinical appearance, and mobility (42). Footpad swelling was quantified in millimeters using a vernier caliper, as described earlier (43, 44).

### Quantification and statistical analysis

All statistical analyses were done using GraphPad Prism software version 10 (GraphPad, La Jolla, USA). Unpaired t-test was performed throughout the study. A p-value of less than 0.05 was considered significant. Results are depicted as median and interquartile ranges. Area under the curve was calculated and IC_50_ titers for each mAb were calculated using the nonlinear regression (curve fit) function in the software.

## Data Availability

The original contributions presented in the study are included in the article/**Supplementary Material**. Further inquiries can be directed to the corresponding authors.
