# Correction: Shared germline-biased plasmablast clonotypes shape early neutralizing antibody responses during acute Chikungunya virus infection

**DOI:** 10.3389/fimmu.2026.1916816

**Published:** 2026-07-02

**Authors:** Kritika Dixit, Kaustuv Nayak, Manpreet Kaur Saini, Sofia Qamar, Prashant Bajpai, Elluri Seetharami Reddy, Sahil Ameer, Vikas Dhar Dubey, Muneeb Pervez, Sagar Kohli, Kamalvishnu Gottimukkala, Shipra Gupta, Naushad Khan, Deepti Maheshwari, Nawaz SM Akhtar, Yadya M. Chawla, Ramesh Chandra Rai, Rohit Sagar, Mohammad Islamuddin, Wajihul Hasan Khan, Nimisha Mishra, Ashok Kumar Patel, Sujatha Sunil, Rakesh Lodha, Vineet Jain, Pratima Ray, Kaja Murali-Krishna, Keshav Saini, Sanjeev Kumar, Anmol Chandele

**Affiliations:** 1 ICGEB-Emory Vaccine Program, International Centre for Genetic Engineering and Biotechnology, New Delhi, India; 2 Kusuma School of Biology Sciences, Indian Institute of Technology, New Delhi, India; 3 Department of Biotechnology, School of Chemical & Life Sciences, Jamia Hamdard, New Delhi, India; 4 Vector-Borne Diseases, International Centre for Genetic Engineering and Biotechnology, New Delhi, India; 5 Department of Pediatrics, All India Institute of Medical Sciences, New Delhi, India; 6 Department of Medicine, Hamdard Institute of Medical Sciences and Research (HIMSR), New Delhi, India; 7 Department of Pediatrics, Division of Infectious Diseases, Emory University School of Medicine, Atlanta, GA, United States; 8 Emory Vaccine Center, Emory University, Atlanta, GA, United States

**Keywords:** acute viral infection, Chikungunya virus, germline antibodies, human monoclonal antibodies, memory B cells, neutralizing antibodies, plasmablasts, single B cell analysis

In the published article, there was an error in the **Funding** statement. The **Funding** statement was displayed as “The author(s) declared that financial support was received for this work and/or its publication. This research was supported by the Indian Government Department of Biotechnology and U.S National Institutes of Health Indo-US Vaccine Action Program, grant number BT/MB/Indo-US/VAP/06/2013; and National Institutes of Health grant 5RO1AI186371.”

The correct **Funding** statement is “The author(s) declare that financial support was received for this work and/or its publication. This research was supported by the Department of Biotechnology, Ministry of Science and Technology, India, grant BT/MB/Indo-US/VAP/06/2013, and the National Institutes of Health, National Institute of Allergy and Infectious Diseases, grant 5R01AI186371.”

