# Supplementary material for: Shared germline-biased plasmablast clonotypes shape early neutralizing antibody responses during acute Chikungunya virus infection
Source: Front Immunol. 2026 Jun 19;17:1846926. doi: 10.3389/fimmu.2026.1846926 (PMC13374033; doi:10.3389/fimmu.2026.1846926)
Supplement: Supplementary file 2 [file SupplementaryFile1.pdf]

Supplementary Table S1: Characteristics of CHIKV confirmed patients from whom PBs were single cell sorted for generation of human monoclonal antibodies.

Characteristics of patients from whom plasmablasts were sorted						
	Patient 1	Patient 2	Patient 3	Patient 4	Patient 5	Patient 6
**Days post onset of clinical symptoms**	2	3	7	7	4	7
**IgM value***	6.33	11.3	23.45	73	44.8	48.9
**IgG value****	2.44	2.7	3.2	26	38.3	13.01
**$PRNT_{50}$****	-	-	-	73	362	490
**% plasmablasts**	4.5%	2.2%	27%	8.5%	13.5%	20.8%
**mAbs made**	13	14	11	21	18	17

** in-house CHIKV specific PCR.*
*** Abcam CHIKV IgM ELISA cut off 11 units.*
**** Abcam CHIKV IgG ELISA cut off 11 units.*

Table S2: Characteristics of seropositive individuals from whom CHIKV-baited memory B cells were single cell sorted for generation of human monoclonal antibodies.

Characteristics of healthy individuals from whom memory B cells were sorted					
	Patient 1$	Healthy 1	Healthy 2	Healthy 3	Healthy 4
**IgM***	14	11.45	16.19	10.08	8.3
**IgG****	22	126.4	35.5	23.7	21.0
**$PRNT_{50}$*****	16166	800	3200	400	71903
**mAbs made**	10	13	2	1	1

** in-house CHIKV specific PCR.*
*** Abcam CHIKV IgM ELISA cut off 11 units.*
**** Abcam CHIKV IgG ELISA cut off 11 units.*
$ paired to patient 1 after 6 months into recovery.

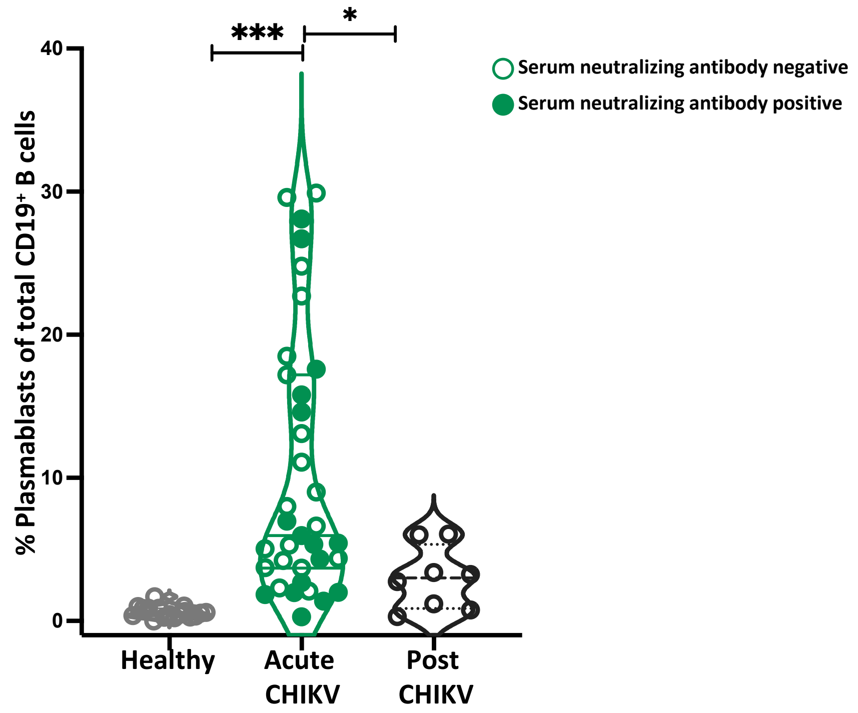

Figure S1: Scatter graph shows frequency of PBs expressed as a percentage of total CD19^{+} B cells in healthy controls (grey), CHIKV patients (green) and post recovery (black). Here, acute CHIKV patients have been indicated that had detectable serum neutralizing antibodies (closed green circles) and those that did not have detectable serum neutralizing antibodies (open green circles).

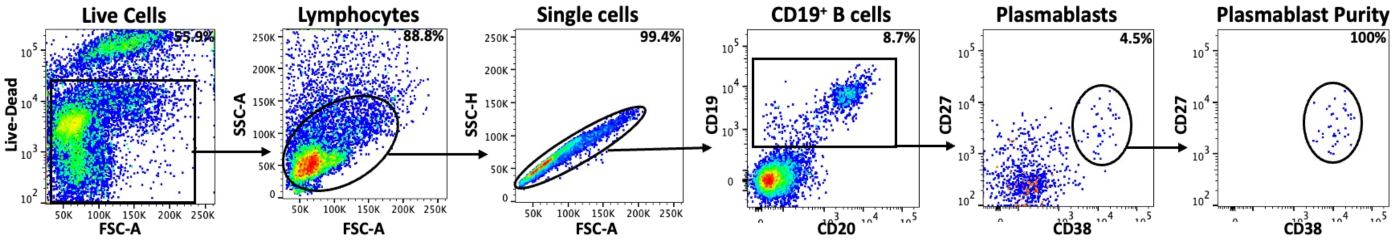

Figure S2: Gating strategy for single-cell sorting of PBs from acute febrile, CHIKV-confirmed patients.

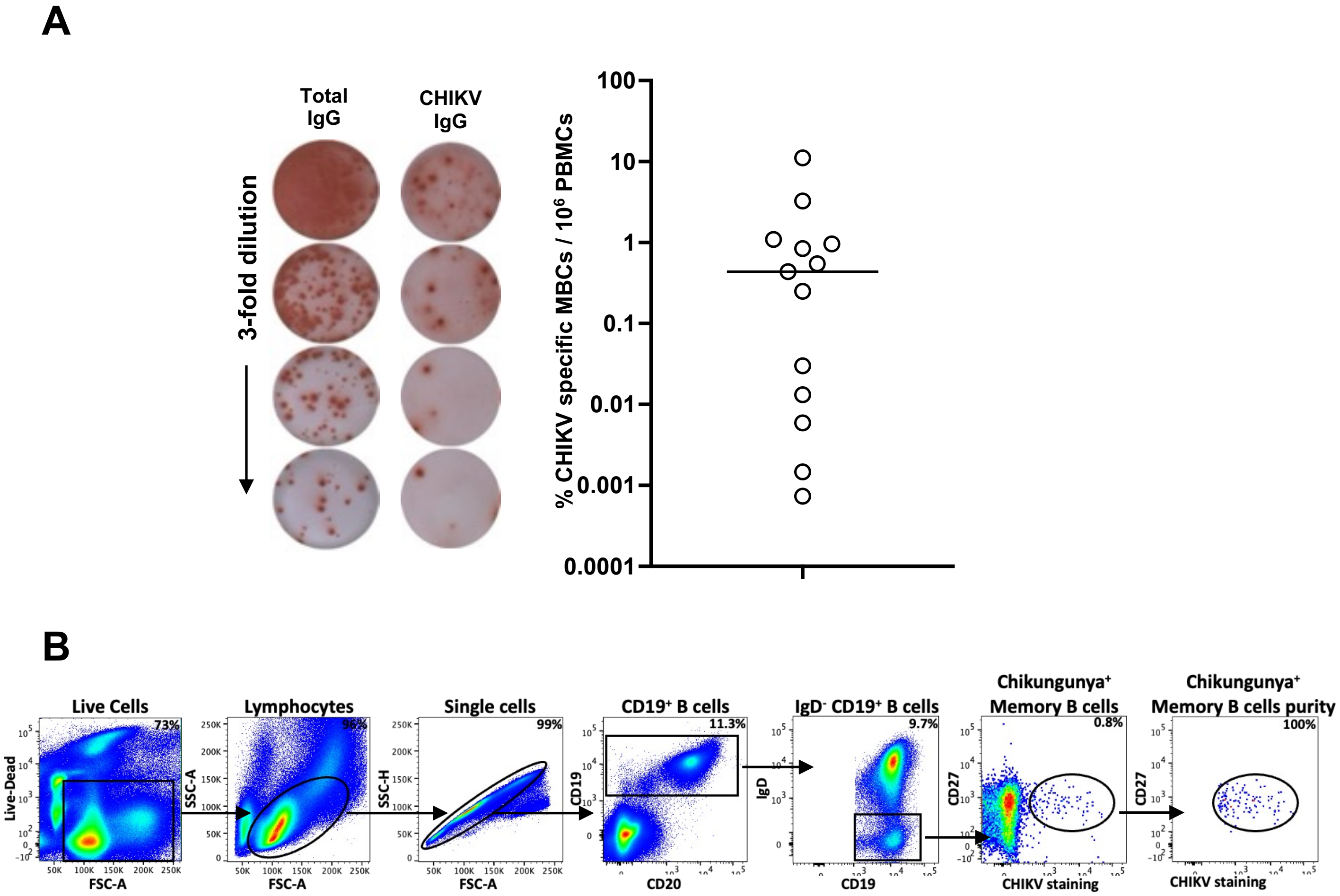

Figure S3: MBC analysis by ELISpot assay and CHIKV-specific sorting of MBCs (A) Schematic illustrating the MBC assay used to quantify total IgG-secreting and CHIKV-specific IgG-secreting B cells. The scatter plot on the right shows the frequency of CHIKV-specific MBCs per 106 PBMCs in CHIKV seropositive individuals. (**B)** CHIKV-baited MBCs from seropositive individuals or CHIKV recovered individuals from which human mAbs were generated.

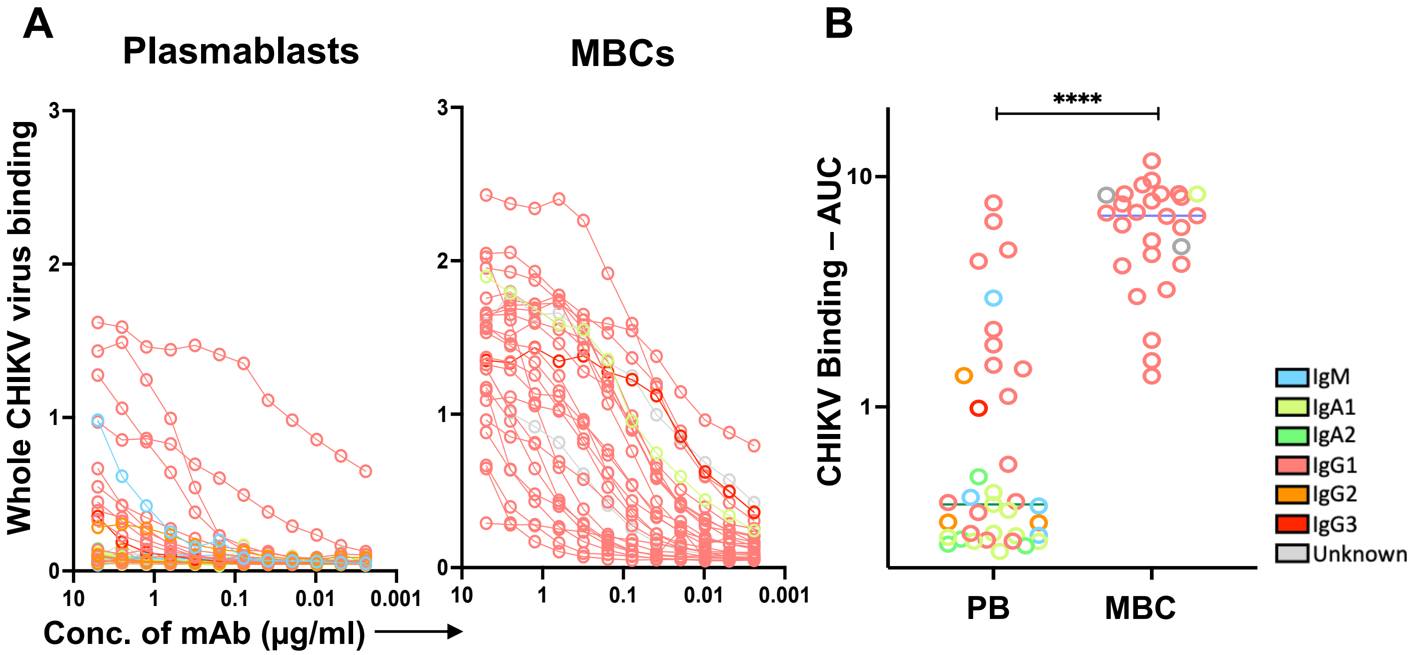

Figure S4: Binding analysis of PB- and MBC-derived mAbs. (A) Line graphs provide isotype distribution of ELISA binding curves of PB-derived (left) and MBC-derived (right) mAbs to plates coated with UV-inactivated CHIKV virions. **(B)** Isotype distribution of the area-under-the-curve (AUC) analysis of ELISA binding responses of PBs and MBC mAbs.

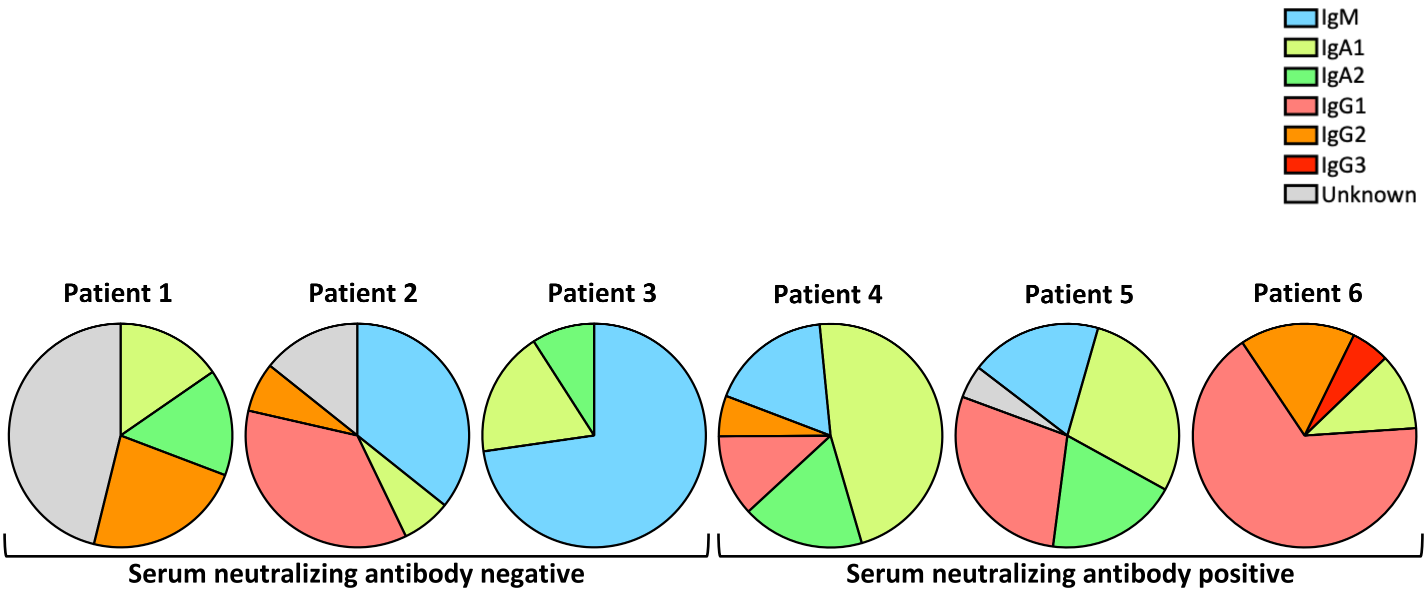

Figure S5: Pie charts show isotype distribution of all mAbs generated from 6 patients from whom plasmablasts were sorted.

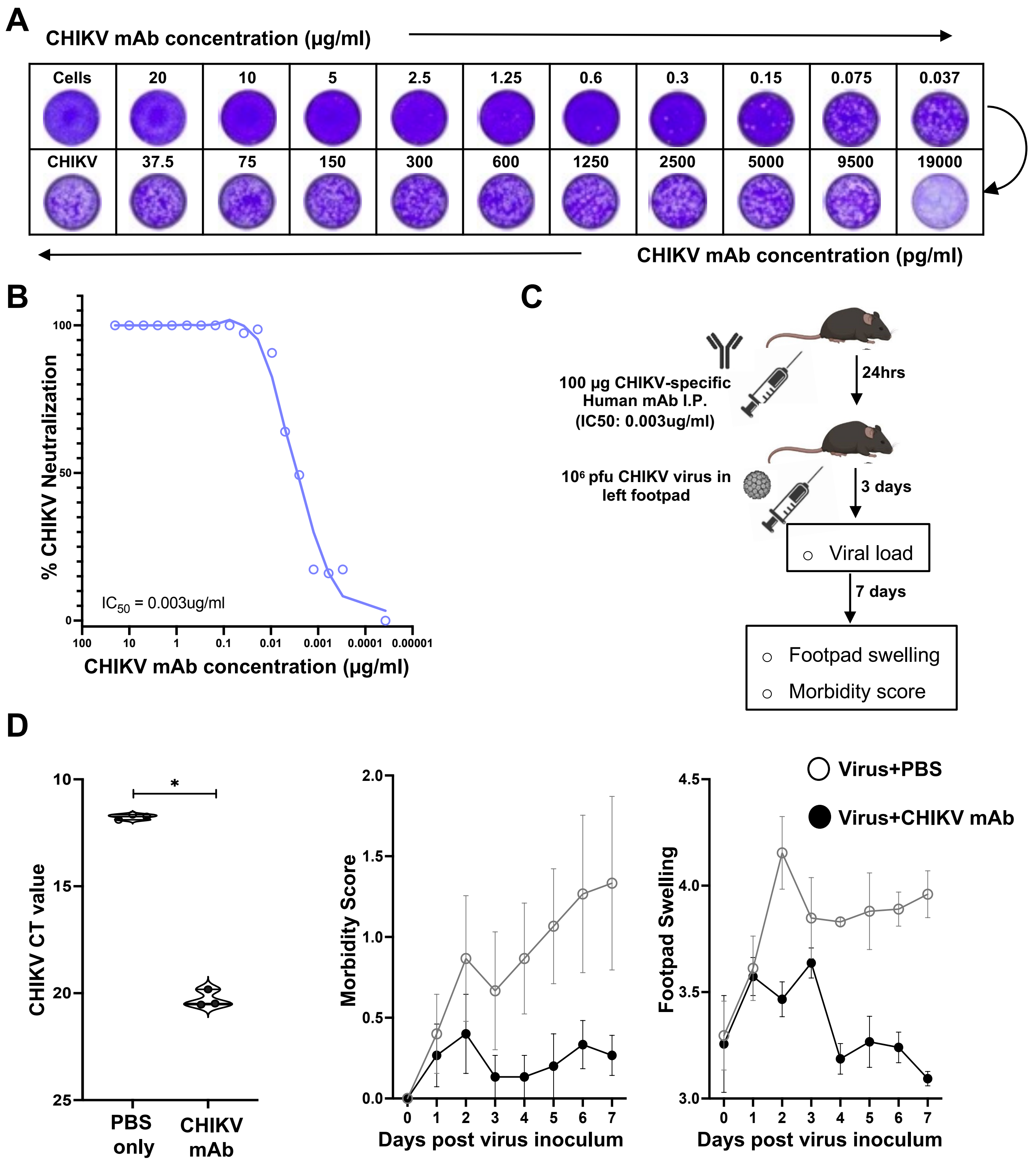

Figure S6: In vivo protective efficacy of a potent human anti-CHIKV monoclonal antibody. (A) Plaque assay provides raw data of CHIKV neutralizing of potent mAbs studied for its in vivo protective efficacy. **(B)** Line graph shows % neutralization (y-axis) at indicated mAb concentrations (x-axis, ug/ml). **(C)** Schematic of the in vivo experimental design. Mice were infected with 10^6 PFU of CHIKV via left footpad inoculation and treated intraperitoneally with a single dose of 100 µg of a CHIKV-specific human mAb (IC_{50} = 0.003 µg/ml) or PBS control. **(D)** Left panel shows CHIKV viral burden measured by RT-PCR (Ct values) in mAb treated and PBS control animals. The middle panel shows composite morbidity scores (y-axis) monitored longitudinally following infection (x-axis) and the right panel shows footpad swelling (y-axis) measured longitudinally following infection (x-axis).

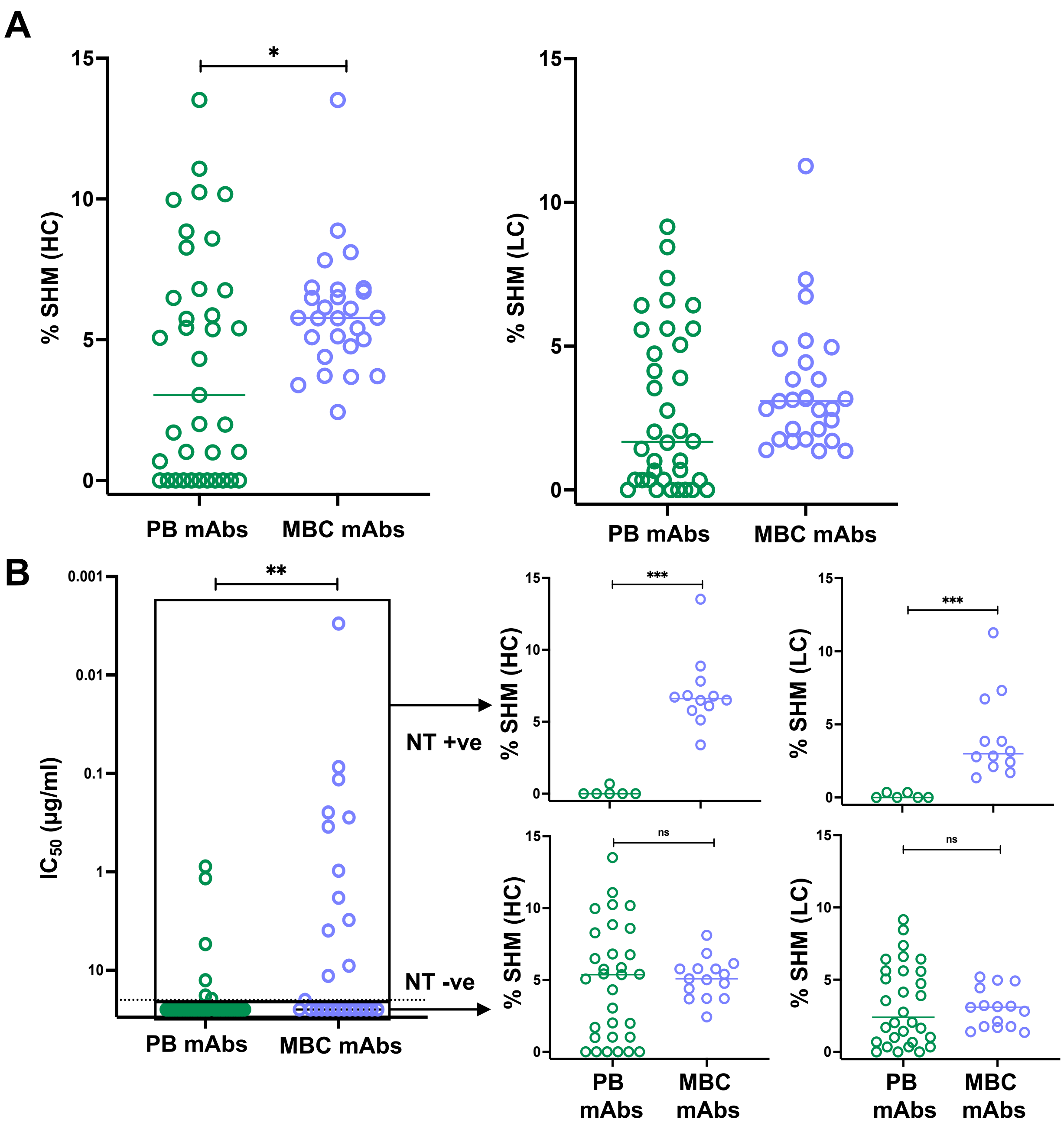

Figure S7: Somatic hypermutations of all CHIKV-reactive antibodies. (A) Scatter graphs show percent somatic hypermutations in the heavy (left) and light (right) chain of all CHIKV reactive mAbs from PB (green, n=37) and MBC (violet, n=27). **(B)** Scatter graph (reproduced from Figure 3C) to delineate neutralizing and non-neutralizing mAbs in both PBs and MBC mAbs. Right panel provides percent somatic hypermutations in heavy (left in right panel) and light (right in right panel) in neutralizing (top row) and non-neutralizing (bottom row) mAbs.

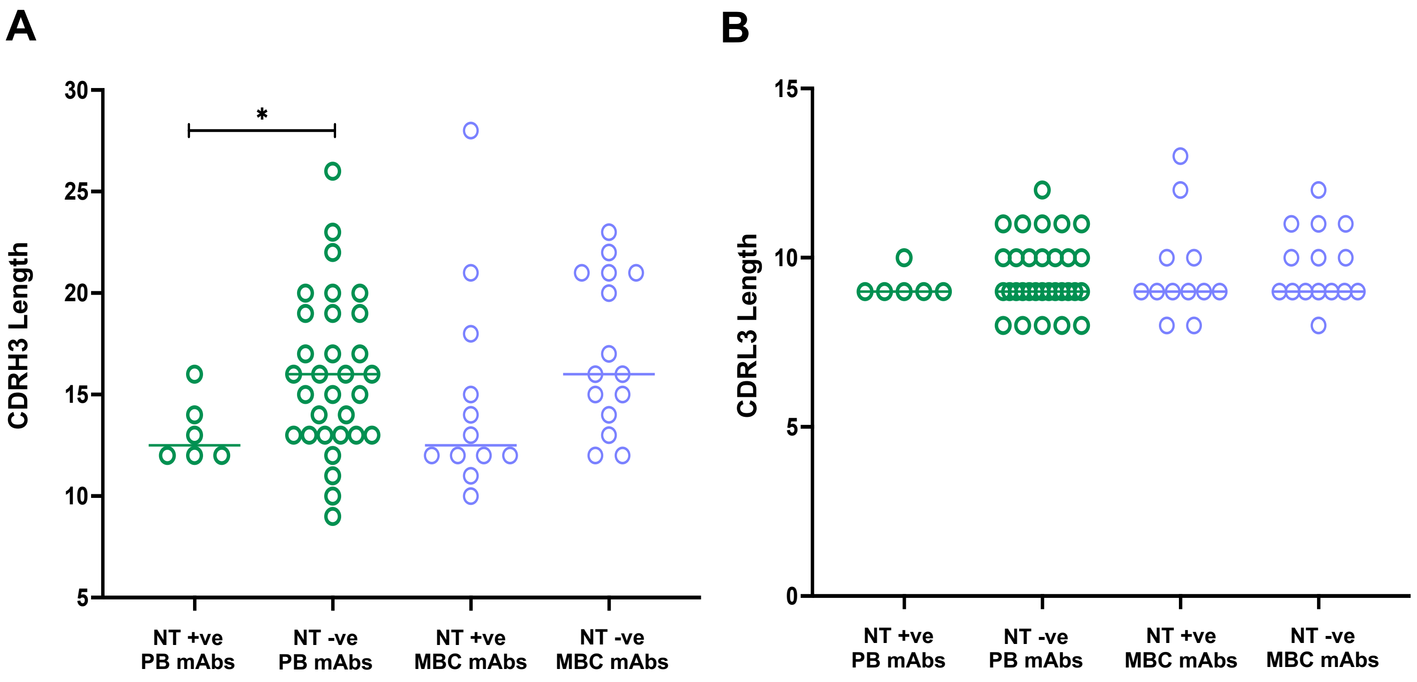

Figure S8: Analysis of CDRH3 and CDRL3 lengths of PB- and MBC-derived nAbs and nAbs. (A) Scatter graph shows differences in CDRH3 lengths in neutralizing and non-neutralizing mAbs from PBs (green) and MBC (blue) mAbs. **(B)** Scatter graph shows differences in CDRL3 lengths in neutralizing and non-neutralizing mAbs from PBs (green) and MBC (blue) mAbs.
